# Multifocal osteolytic lesions of the skull: a primary cavernous hemangioma mimicking a neoplastic invasive lesion

**DOI:** 10.7603/s40681-015-0012-y

**Published:** 2015-06-09

**Authors:** I-Han Hsiao, Der-Yang Cho, Chun-Lin Liu

**Affiliations:** 1 Medical Department Department of Neurosurgery, China Medical University Hospital, No. 2, Yu-Der Road, 404 Taichung, Taiwan; 2 Medical Department, China Medical University, 404 Taichung, Taiwan

**Keywords:** Cavernous hemangioma, Langerhans cell, histiocytosis, Skull, Osteolytic Lesions, Intraosseous tumor

## Abstract

Intraosseous cavernous hemangioma is a rare cause of osteolytic lesions of the skull, and its multifocal type is even more infrequent. This tumor is difficult to accurately diagnose by imaging and can be confused with osteolytic Langerhan’s cell histiocytosis or other neoplasms. Here we present a case of multifocal intraosseous cavernous hemangioma of the skull treated with surgical intervention in our hospital five years ago. A review of related literatures and case reports is also provided to help clarify the diagnosis and devise treatment regimens. In light of the difficulties of early diagnosis, early en bloc surgical removal is recommended.

## 1. Introduction

Primary cavernous hemangiomas are rare skeletal tumors accounting for 0.7-1% of all bone neoplasms [4]. These tumors, which arise from the intrinsic vasculature are mostly found in vertebral bodies. Cavernous hemangiomas are usually unifocal, and they represent 0.2% of all benign neoplasms of the skull. The majority of these lesions are asymptomatic, but patients can present with focal pain or palpable mass. A multifocal osteolytic lesion can initially be confused with Langerhan’s cell histiocytosis (LCH) or a malignant neoplasm. Here we present a case of multifocal cavernous hemangiomas of the skull bone.

## 2. Case presentation

**Fig. 1:**
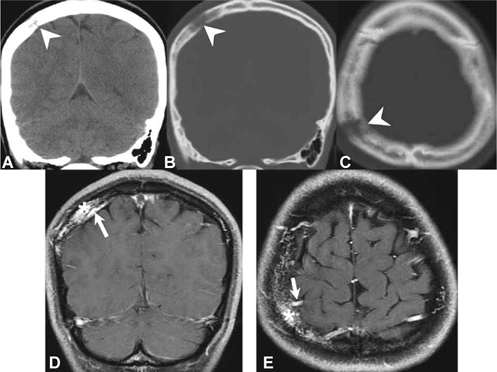
(A-C) A parietal osteolytic lesion on brain CT. (D, E) The enhanced lesion (asterisk) with a feeding artery (arrow) revealed by gadolinium-enhanced T1-weighted imaging.

**Fig. 2:**
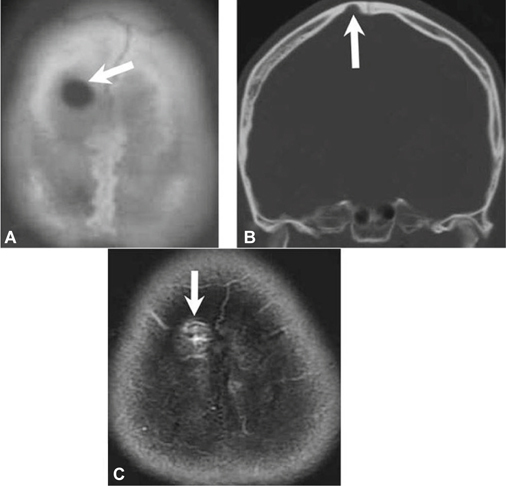
(A, B) Another osteolytic lesion at the frontal area on brain CT. (C) The enhanced lesion (arrow) revealed by gadolinium-enhanced T1-weighted imaging.

**Fig. 3:**
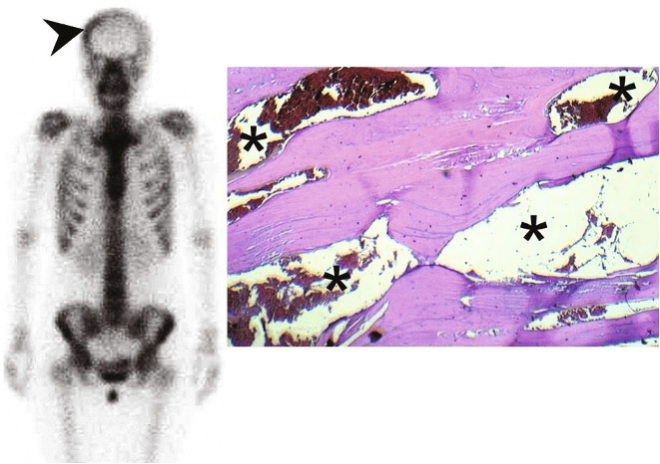
Left: A bone scintigraphy image. The arrow-head indicates right skull bone enhancement. Right: The histologic examination showed a cavernous hemangioma (black asterisk) of the diploe with thin-walled, dilated capillary spaces lined by with endothelial cells. No malignancy was observed.

A 29-year-old female came to our neurology outpatient clinic because of a painful skull defect found incidentally over the right parietal area. The lesion was soft and with mild dimpling. Intermittent pain had started at least 3 weeks before the initial visit. She denied any history of head injury or systemic disease. Cranial computed tomography (CT) and magnetic resonance imaging (MRI) identified 2 individual osteolytic lesions with contrast enhancement (Figures 1, 2) over the right parietal (10 mm * 9 mm) and frontal (8 mm * 9 mm) areas of the skull. In particular , the CT scan revealed osteolytic lesions with erosion of the skull bone, whereas MRI showed low signals on T1-weighted images, high signals on T2-weighted images, and heterogeneous enhancing effects on gadolinium-enhanced T1-weighted images. A neoplastic invasive lesion, such as LCH or malignant metastasis tumor, was initially suspected. A series of tests including: L-spine MRI, bone scintigraphy (Figure 3), and analysis of tumor markers did not reveal abnormal results. A large craniectomy was performed for the evacuation of the 2 osteolytic lesions, and cranioplasty with polymethylmethacrylate was carried out for skull reconstruction. The dura was intact, but diffused oozing and central hyperemia were noted during the surgery. The final histological report confirmed the diagnosis of intraosseous cavernous hemangioma. The patient recovered well. She has been followed up for 4 years with no recurrence.

## 3. Discussion

Primary intraosseous cavernous hemangioma is a rare, benign, and slow-growing tumor formed by blood vessels separating fibrous tissue and accounting for 0.2% of all benign tumors of the skull [4]. In the skull, these tumors are fed by the branches of the external carotid artery, especially the superficial temporal artery, posterior occipital artery, and branches of the middle meningeal artery [5]. Cavernous hemangiomas are usually asymptomatic lesions, but the clinical presentation can include cosmetic changes, increased intracranial pressure caused by parenchymal compression, and the cranial nerve deficits [3]. The peak age of skull hemangiomas is the fourth decade of life, and women are affected 2-4 times more often than men [6]. Some reported treatments include curettage and radiotherapy [2]. Curettage may lead to massive perioperative bleeding and recurrence after the operation. Radiotherapy can only can prevent the tumor from growing, but it cannot eradicate the lesions. Additionally, there are reports describing malignant transformation of intraosseous cavernous hemangiomas after radiotherapy.

Differential diagnoses of intraosseous neoplasm include LCH, osteoma, sarcoma, and fibrous dysplasia. LCH is caused by abnormal proliferation of dendritic cells, which often occurs in young patients. It is commonly located in the skull , especially in the parietal and frontal bones [7]. Compared to LCH, the great majority of the reported cases of skull hemangiomas are unifocal, although multiple hemangiomas have also been described [6]. Wyke reported that 7 out of 40 reported cases of primary hemangioma of the skull were multifocal [9]. According to the study performed at Mayo Clinic in 1975, only 2 out of 43 patients with hemangiomas had multiple lesions [8].

Our literature search failed to identify an ideal noninvasive method that can differentiate cavernous hemangioma from other diseases such as LCH or malignant cell invasions. Therefore, in the majority of cases, the final diagnosis is established only after surgery. For the above reasons, early en bloc surgical removal of intraosseous tumors is recommended irrespectively of whether the osteolytic lesion is benign or malignant , and unifocal or multifocal. Correction of cosmetic changes and treatment of local pain are other indication for the surgery [3].
